# Congenital hyperinsulinism in an individual with CHARGE syndrome and a pathogenic *CHD7* variant

**DOI:** 10.1210/jcemcr/luag016

**Published:** 2026-02-24

**Authors:** Consuelo Ibeas, Franco Giraudo, Jonna M E Männistö, Sarah E Flanagan, Verónica Mericq

**Affiliations:** Institute of Maternal and Child Research, University of Chile, Santiago 8360160, Chile; Pediatrics Department, Clinical Hospital San Borja Arriarán, Santiago 8360160, Chile; Institute of Maternal and Child Research, University of Chile, Santiago 8360160, Chile; Pediatrics Department, Clinical Hospital San Borja Arriarán, Santiago 8360160, Chile; Department of Clinical and Biomedical Science, University of Exeter, Exeter EX2 5DW, UK; Kuopio Pediatric Research Unit (KuPRu), University of Eastern Finland, Kuopio FI-70211, Finland; Department of Clinical and Biomedical Science, University of Exeter, Exeter EX2 5DW, UK; Institute of Maternal and Child Research, University of Chile, Santiago 8360160, Chile

**Keywords:** hyperinsulinemic hypoglycemia, hyperinsulinism, CHARGE syndrome

## Abstract

Hyperinsulinemic hypoglycemia (HI) is a rare feature in individuals with coloboma, heart defects, atresia choanae, retardation of growth and development, genital abnormalities, and ear abnormalities (CHARGE) syndrome, though its underlying mechanisms remain poorly understood. We report a Chilean female proband with genetically confirmed CHARGE syndrome caused by a pathogenic variant in the *CHD7* gene, who presented with HI in the neonatal period. Initial hypoglycemia was detected on days 2-3 of life, followed by recurrent episodes prompting biochemical investigation. On day 21, HI was biochemically confirmed. Comprehensive hormonal evaluation, including cortisol and growth hormone testing, excluded deficiencies in these hormones as contributing factors. Genetic screening of 22 known HI-associated genes revealed no pathogenic variants, supporting the hypothesis that HI in this case is related to CHARGE syndrome rather than being a coincidental finding. The patient responded well to diazoxide treatment, which allowed for maintenance of normoglycemia, with gradual dose reduction as glucose management normalized. This case, along with 2 previously reported cases, suggests that HI can be an integral part of CHARGE syndrome. Further research is needed to understand the mechanisms connecting *CHD7* variants and HI and to refine management strategies for affected individuals.

## Introduction

CHARGE syndrome (named after the acronym coloboma, heart defects, atresia choanae, retardation of growth and development, genital abnormalities, and ear abnormalities) is a complex, multisystem disorder affecting 1 in 10 000-15 000 live births. It is primarily caused by heterozygous dominant, loss-of-function variants in the *CHD7* gene, which encodes chromodomain helicase DNA-binding protein 7, a key regulator of transcription during embryonic development (OMIM #214800) [1].

Persistent hypoglycemia has been reported as a feature of CHARGE syndrome, which can present in the early neonatal period and be transient or persist into childhood [2-4]. The underlying cause of hypoglycemia can be attributed to coexisting endocrine disorders or may result from feeding difficulties due to cranial nerve dysfunction and swallowing impairments [2-5]. In one previously reported individual, persistent hypoglycemia was attributed to growth hormone (GH) deficiency concomitant with hyperinsulinism [4]. In another case, persistent hypoglycemia was due to central adrenal insufficiency with some degree of hyperinsulinism, indicated by low ketones and high glucose intake, without requiring specific hyperinsulinemic hypoglycemia (HI) therapy [2].

We now describe a third case of a newborn with CHARGE syndrome who experienced recurrent episodes of HI persisting through the first 3 years of life. This observation further supports an association between the 2 conditions and highlights the importance of considering hypoglycemia screening in neonates with CHARGE syndrome.

## Case presentation

The Chilean female proband was born via cesarean section due to breech presentation, to healthy, nonconsanguineous parents. Complications during the pregnancy included diet-controlled gestational diabetes and an Ebstein anomaly detected prenatally and confirmed postnatally with an echocardiogram. At birth, her size was appropriate for gestational age, but Apgar scores were low (3 at 1 minute and 6 at 5 minutes). The first capillary glucose measured 4 hours after birth was normal at 52 mg/dL (Standard International [SI]: 2.9 mmol/L).

On the second and third days of life, asymptomatic episodes of hypoglycemia (lowest recorded glucose: 31 mg/dL [SI: 1.7 mmol/L]) were observed in association with feeding difficulties and inadequate oral intake. Intravenous glucose infusion was initiated at a maximum glucose infusion rate (GIR) of 8 mg/kg/min, followed by nasogastric tube feeding with breast milk, allowing gradual reduction of the GIR over the following days.

Between days 7 and 19, total parental nutrition was started following surgical closure of a patent ductus arteriosus due to pulmonary overflow. During this period, hydrocortisone (50 mg/m^2^/day) was administered for suspected critical illness–related corticosteroid insufficiency based on clinical context. On day 19, parenteral nutrition was transitioned to enteral feeding (77 mL/kg/day) via nasogastric tube.

On day 21, asymptomatic episodes of hypoglycemia (32-57 mg/dL [SI: 1.8-3.2 mmol/L]) recurred, prompting the reinitiation of glucose infusion at a maximum rate of 6 mg/kg/min. Combined with enteral nutrition, this provided a total glucose intake of 9.8 mg/kg/min.

## Diagnostic assessment

The results of a critical sample (blood and urine samples collected at the time of documented hypoglycemia [plasma glucose <50 mg/dL] for etiologic evaluation of hypoglycemia) met the diagnostic criteria of HI, including suppressed ketogenesis, inappropriate insulin secretion, and marked response to glucagon (Table 1) [6]. A second critical sample obtained when the blood glucose level was above the hypoglycemic threshold (59 mg/mL [SI: 3.3 mmol/L]) revealed a relatively low GH level and serum cortisol levels that, together with the clinical history, led to the initiation of hydrocortisone at a physiological dose (15 mg/m^2^/day). No further confirmatory tests for adrenal insufficiency were performed, but additional endocrine evaluations showed normal insulin-like growth factor 1 (IGF-1) and insulin-like growth factor binding protein 3 (IGFBP-3), ruling out GH deficiency, normal thyroid function, and normal metabolic workup, including amino acid and acylcarnitine profiles, ammonium levels, and blood gases.

**Table 1:** Summary of clinical characteristics at birth, follow-up, and laboratory test results

Clinical characteristics	
Variant	*CHD7* c.7879C>T (p.Arg2627*^a^*)
Sex	Female
Gestational age	38 weeks
Birth weight (SDS*^a^*)	2990 g (−0.5)
Birth length (SDS*^a^*)	47 cm (−1.3)
Birth head circumference (SDS*^a^*)	35 cm (+0.3)
Diagnosis of HI	
Age at detection of hypoglycemia	2 days
Age at diagnosis of HI	21 days
Glucose	30 mg/dL (1.6 mmol/L) ↓
	(reference range: >50 mg/dL [SI: >2.8 mmol/L])
Insulin	3.7 μIU/mL (22.2 pmol/L) ↑
	(expected during hypoglycemia to be undetectable)
β-Hydroxybutyrate	Undetectable ↓
Maximum GIR	8 mg/kg/min ↑
Glucose test (0.1 mg/kg IM), increase in glucose level after 30 minutes	+46 mg/dL; from 50 to 96 mg/dL ↑
	(SI: +2.5 mmol/L; from 2.8 to 5.3 mmol/L)
	(marked glycemic response defined as an increase >30 mg/dL (SI: >1.7 mmol/L)
Additional biochemistry during hypoglycemia	
Ammonia	51.3 μg/dL (30.1 µmol/L) ↔
	(normal value: <170 μg/dL [SI: <99.8 µmol/L])
Lactate	11.3 mg/dL (1.3 mmol/L) ↔
	(reference range: 9.9-31.5 mg/dL [SI: 1.1-3.5] mmol/L)
GH	2.1 ng/mL (2.1 µg/L) ↓
	(expected during hypoglycemia >7 ng/mL [SI: >7 µg/L])
IGF-1	65.1 ng/mL (8.5 nmol/L) ↔
	(reference range: 15-109 ng/mL [SI: 2.0-14.3 nmol/L])
IGFBP-3	1725 ng/mL (225.5 nmol/L) ↔
	(reference range: 1000-3000 ng/mL [SI: 130.7-392.2 nmol/L])
Cortisol	5.6 μg/dL (154.5 nmol/L) ↓
	(expected during hypoglycemia >18 μg/dL [SI: >496.5 nmol/L])
TSH	6.6 mIU/mL (6.6 µIU/mL) ↔
	(reference range for newborns: 0.43-16.1 mIU/mL [SI: 0.43-16.1 µIU/mL])
fT4	1.6 ng/dL (20.6 pmol/L) ↔
	(reference range: 0.8-3.1 ng/dL [SI: 10.7-39.9 pmol/L])
Treatment for HI	
Age at initiation of diazoxide	40 days
Initial dose of diazoxide	10 mg/kg/d
Syndromic features	
Dysmorphology	At birth: Sparse eyebrows, horizontal palpebral fissures, short columella, downturned mouth, rotated ears with sparse lobes and a prominent antihelix, short and broad neck, low hair implantation, a wide shield chest, sharp fingers with finger webs on the left side. Facial asymmetry (when crying) consistent with left orbicularis hypoplasia. Normal skull configuration, mammary glands, and genitalia. Later: coarse features.
Structural abnormalities	Severe Ebstein anomaly
Other features	Physical examination: Bilateral optic nerve coloboma, bilateral sensorineural hearing loss, low muscle mass.
	Brain magnetic resonance imaging:
	Bilateral vestibular dysplasia. Right cochlear opening stenosis, absence of left cochlear opening, and cochlear nerves not visualized. Bilateral colobomas. Platybasia. Right inferior parietal and paramedian thalamic infarction, reduction of white matter volume, small subdural hematomas, and bilateral subdural hygromas.

The arrows denote an alteration and its direction or a normal expected level.

Abbreviations: fT4, free thyroxine; GH, growth hormone; GIR, glucose infusion rate; HI, hyperinsulinemic hypoglycemia; IGF-1, insulin-like growth factor 1; IGFBP-3, insulin-like growth factor binding protein-3; IM, intramuscular; SDS, standard deviation score; TSH, thyroid-stimulating hormone.

^*a*^According to WHO Child Growth Standards (0-5 years).

The patient was found to have bilateral optic nerve coloboma, bilateral sensorineural hearing loss, and several central nervous system abnormalities, which were identified through brain magnetic resonance imaging. These clinical and imaging findings prompted genetic testing that was performed by INVITAE®, which revealed a pathogenic heterozygous stop-gain variant in the *CHD7* gene (c.7879C>T; p.Arg2627*) (GRCh37, NM_017780.4).

The child met diagnostic criteria for CHARGE syndrome, fulfilling 3 major (coloboma, ear abnormality, and pathogenic *CHD7* variant) and 4 minor criteria (cranial nerve dysfunction and hearing loss, feeding difficulties, structural brain anomalies, and heart malformation) [7]. No evidence of developmental delay, pituitary dysfunction, or renal, skeletal, limb, or genital abnormalities was observed.

## Treatment

Reflecting the complexities of a situation involving multiple conditions, the initiation of HI medication was delayed during the hospitalization due to a partial response to hydrocortisone, the need for intercurrent cardiac surgeries, and the time taken to obtain the 2 critical samples fulfilling the diagnostic criteria for HI. Diazoxide (10 mg/kg/day) was subsequently initiated on day 48 of life, resulting in an excellent response and maintenance of normoglycemia with total enteral nutrition (140 mL/kg/day). Preprandial glucose levels remained stable, and the patient was discharged. It remains unclear whether a formal fasting test was performed.

## Outcome and follow-up

A summary of the clinical details at follow-up is presented in Table 2. At the age of 2.3 years, the child was referred to our endocrinology team due to ongoing asymptomatic hypoglycemia while receiving diazoxide (13 mg/kg/day). She had been weaned off hydrocortisone by the age of 2 years. However, continuous glucose monitoring (CGM) using FreeStyle Libre® between the ages of 2.3 and 2.8 years revealed no periods of hypoglycemia. On contrary, the CGM data showed elevated glucose levels (>150 mg/dL [SI: 8.3 mmol/L]), often occurring about 1-hour postfeed. Glucose levels were >180 mg/dL (SI: >10.0 mmol/L) ∼20% of the time [2]. Despite these findings, we only began to slowly reduce the diazoxide dose from 12.7 mg/kg/day at 2.8 years of age due to parental concerns about potential hypoglycemia during recovery from cardiac surgery. The patient was receiving a combination of enteral nutrition (180 mL of milk powder 3 times daily) and gastrostomy tube feeding (2 times daily with fruit-based foods). Additionally, at 2.8 years, the patient developed postnatal growth deficiency, generalized hypertrichosis (likely associated with diazoxide use), and low muscle mass.

**Table 2:** Details of treatment, growth, and biochemical data during follow-up

Latest follow-up at 3.3 years of age	
Diazoxide dose	7.5 mg/kg/d
HbA1c	5.8% (40 mmol/mol)
	(reference range: 4.5-5.7% [SI: 26-39 mmol/mol])
Fasting morning cortisol	8.2 µg/dL (226 nmol/L)
	(reference range: 5-25 µg/dL [SI: 140-690 nmol/L])
β-Hydroxybutyrate	< 0.6 mmol/L (reference range: < 0.6 mmol/L)
Follow-up between 2.3 and2.8 years of age	
Diazoxide dose	12.7 mg/kg/d
HbA1c	6.1% (43 mmol/mol)
	(reference range: 4.5-5.7% [SI: 26-39 mmol/mol])
Typical summary on continuous glucose measurement	Mean glycemia 133 mg/dL (7.4 mmol/L)
	Estimated HbA1c 6.5% (48 mmol/mol)
	Time in range:
	<1% ≤54 mg/dL (≤3 mmol/L)
	5% within 55-69 mg/dL (3.0-3.8 mmol/L)
	20% >180 mg/dL (>10 mmol/L)
Glucose test (0.1 mg/kg IM), increase in glucose level after 30 minutes	+111 mg/dL; from 35 to 146 mg/dL ↑
	(+6.3 mmol/L; from 1.9 to 8.2 mmol/L)
	(marked glycemic response defined as an increase >30 mg/dL [(SI: >1.7 mmol/L])
Auxological measurements at 2.8 years of age	
Weight (SDS*^a^*)	10.7 kg (−2.28)
Height (SDS*^a^*)	86 cm (−1.98)
Weight for height percentile (SDS*^a^*)	8th (−1.39)
Head circumference (SDS*^a^*)	47.5 cm (−0.65)

Abbreviations: HbA1c, hemoglobin A1c; IM, intramuscular; SDS, standard deviation score.

^a^According to WHO Child Growth Standards (0-5 years).

At 3 years old, she had a marginally elevated glycosylated hemoglobin (HbA1c) of 5.7% (SI: 39 mmol/mol) (reference range: 4.5-5.7% [SI: 26-39 mmol/mol]), thyroid-stimulating hormone (TSH) of 2.5 mIU/L (SI: 2.5 μIU/mL) (reference range: 0.7-5.97 mIU/L [SI: 0.7-5.97 μIU/mL]), free thyroxine (FT4) of 1.7 ng/dL (SI: 21.9 pmol/L) (reference range: 0.8-3.1 ng/dL [SI: 10.7-39.8 pmol/L]), and a normal IGF-1 of 114 ng/mL (SI: 14.9 nmol/L) (normal value 26-155 ng/mL [SI: 3.4-20.3 nmol/L]). At the most recent follow-up at 3.3 years, the child was on a diazoxide dose of 7.5 mg/kg/day, without symptoms or objectively proven hypoglycemia. She had an HbA1C of 5.8% (SI: 40 mmol/mol), fasting morning cortisol 8.2 µg/dL (SI: 226.2 nmol/L) (reference range: 5-25 µg/dL [SI: 140-690 nmol/L]) and capillary blood ketones <0.6 mmol/L (reference range: <0.6 mmol/L). The child is continuing on a diazoxide reduction plan.

To investigate whether the hypoglycemia observed in this child was likely related to the *CHD7* disorder or was coincidental, next-generation sequencing of the known HI genes was performed at the Exeter Genomics Laboratory (UK) using previously described methods [8]. This analysis excluded single-nucleotide variants and copy number variants in 22 HI genes (*ABCC8, AKT2, CACNA1D, CREBBP, EP300, FOXA2, GCK, GLUD1, GPC3, HADH, HNF1A, HNF4A, INSR, KCNJ11, KDM6A, KMT2D, MAFA, NSD1, PHOX2B, PMM2, SLC16A1,* and *TRMT10A*).

## Discussion

We describe a child with genetically and clinically confirmed CHARGE syndrome resulting from a pathogenic *CHD7* variant. The child experienced hypoglycemia first noted on days 2-3 of life. Hyperinsulinemic hypoglycemia was biochemically confirmed on day 21 with the detection of insulin during hypoglycemia, absence of ketones, and a positive glycemic response to glucagon.

While the precise mechanisms behind the HI have not been fully elucidated in this syndrome, this report adds to the growing body of evidence suggesting that HI can be a feature of CHARGE syndrome, bringing the total number of reported cases to 3 [2-4]. Transient hypoglycemia has also been reported in multiple cases [3, 9], but interestingly, at least one of these children had ketones detected at the time of hypoglycemia, which ruled out HI [9]. This observation aligns with previous reports suggesting that there are multiple biological and/or genetic mechanisms that can lead to hypoglycemia in these patients [2, 5], which is similar to other syndromic conditions, such as Beckwith–Wiedemann syndrome, Kabuki syndrome, and trisomy 21 [2, 10].

In the 2 published cases of CHARGE syndrome with suggested HI, the hypoglycemia was thought to result from different causes: GH deficiency in one case and dysfunction of the hypothalamic–pituitary–adrenal axis in the other [2, 4]. The latter case had low ketones at day 2 of life but as a critical sample during hypoglycemia was not reported, an incidental finding related to early life cannot be ruled out [11]. This highlights the complexity in determining causal relationships between the clinical features of CHARGE syndrome and hypoglycemia. Further complicating this issue, it has been shown that low GH or adrenocorticotropic hormone/GH levels at the time of hypoglycemia do not necessarily imply deficiency of these hormones in neonates with HI or hypoglycemia [12, 13]. Although the present case did not undergo confirmatory testing for critical illness–related cortisol insufficiency, the available evidence suggests this is unlikely to have contributed significantly to the hypoglycemia. Specifically, the adequate cortisol level (>18 μg/dL [SI: >496.5 nmol/L]) in the second critical sample [14] and a recent fasting cortisol in the normal range argue against significant adrenal insufficiency. Additionally, normal IGF-1 and IGFBP-3 levels and the absence of pituitary anomalies on magnetic resonance imaging further suggest that GH deficiency was not a contributing factor at the time of hypoglycemia onset. These findings emphasize the need for careful and comprehensive evaluation of hormone levels, as hypoglycemia in syndromic conditions like CHARGE syndrome can have multifactorial causes.

To help exclude the possibility of HI being coincidental in our patient, we performed a comprehensive screening of the known HI genes. While it is recognized that up to 50% of children with persistent HI may not have a genetic diagnosis following routine screening [15], the absence of a disease-causing variant in the 22 genes tested lends support that the CHARGE syndrome and HI in our patient are likely to be related rather than coincidental.

The overall natural history of HI of this patient was quite typical of other diazoxide-responsive forms of HI, demonstrating a reduced severity that allowed for ongoing dose reduction in early childhood [16, 17]. Our patient initially required a relatively high dose of diazoxide, which was similar to the individuals with CHARGE syndrome reported by Sekiguchi et al [4] (maximum of 13 and 11 mg/kg/day, respectively). However, the other reported individual did not require HI medication [2]. Future follow-up and a fasting test after the potential trial of weaning off from diazoxide will help confirm whether complete remission has been achieved in this patient, as highly expected.

## Learning points

- HI, though rare, may be a feature of CHARGE syndrome.
- CHARGE syndrome should be considered in neonates with congenital anomalies and persistent hypoglycemia.
- Comprehensive genetic testing helps exclude alternative causes and strengthens the association between CHARGE syndrome and HI.

## Data Availability

Data sharing is not applicable to this article as no datasets were generated or analyzed during the current study.
